# Sensory Analysis Performed within Augmented Virtuality System: Impact on Hedonic Scores, Engagement, and Presence Level

**DOI:** 10.3390/foods13152456

**Published:** 2024-08-03

**Authors:** José Carlos Ribeiro, Célia Rocha, Bruna Barbosa, Rui Costa Lima, Luís Miguel Cunha

**Affiliations:** 1 GreenUPorto/INOV4Agro & DGAOT, Faculty of Sciences of the University of Porto, Rua da Agrária, 747, 4485-646 Vairão, Portugal; 2 Sense Test, Lda, Rua Zeferino Costa, 341, 4400-345 Vila Nova de Gaia, Portugal

**Keywords:** consumer, context, hedonic discrimination, immersive environment, mixed reality, virtual reality

## Abstract

Sensory analysis methodologies are performed in sensory booths designed to minimise external stimuli, lacking ecological validity. Immersive environments are used to introduce contextual cues, but there is a lack of studies using mixed reality systems. The main goal of this study was to evaluate an augmented virtuality (AV) system where participants are inserted into a virtual environment and evaluate a real product, being able to interact with both dimensions. A panel of 102 consumers evaluated five samples of commercial peach nectars in three sessions, each in a different environment: public food court, living room (AV environments), and laboratory (traditional sensory booth). Consumers rated overall liking, followed by open comments, and also answered an Engagement (EQ) and a Presence Questionnaire (PQ). The type of environment only affected hedonic discrimination among samples, with the laboratory setting being the only one with sample discrimination. Nonetheless, each sample was not evaluated differently across the different environments. Concerning engagement, the environment only significantly influenced the EQ’s ‘Affective Value’ factor, being higher when using an AV system. The level of presence in the virtual environment was significantly higher in the public food court, being significantly correlated with the EQ factor scores.

## 1. Introduction

The food choice process is a complex behaviour that has gained recognition in sensory and consumer science, leading numerous researchers to dedicate themselves to its study over the past few decades. Several key factors influence the determinants of food acceptance and choice [1], but in the last few years, context and its influence on consumers’ behaviour and food/beverage perception have been identified as two of the most important research topics [2,3,4]. Contextual cues, such as eating location [5], social context [6], or sound [7], can have a significant impact on consumer perception and behaviour. It is important to note that the interaction between the product and the consumption environment can also influence acceptance [6,7] and that some products are more susceptible to contextual factors than others [8].

The classical methodologies of sensory analysis and consumer perception are usually performed in laboratories intentionally designed to suppress external factors and are standardised in aspects such as colour, temperature, or lighting [9]. Performing evaluations in these controlled environments ensures that the observed effect can be attributed to experimental variables rather than environmental factors. These testing conditions increase consumers’ ability to identify differences between products [10] while also allowing researchers to control experimental conditions and assuring both time and cost-effectiveness [11]. These experiments present high internal reliability, but the lack of contextual and environmental cues severely hampers ecological and external validity [3,12]. Furthermore, experiments in such controlled environments can also lead to a lack of engagement from consumers due to boredom [13,14]. The lack of contextual information in these evaluation locations leads to inaccurate predictions of food-related behaviours [15] and has been referenced as one of the main factors for the inability of consumer methodologies to predict the success of new products on the market [16,17].

More ecologically validated results can be obtained by testing products in contexts similar to those where they are usually consumed, and the more engaging the context, the closer a participant’s responses will be to those obtained in the real world [18]. Immersive technologies have increasingly been used to recreate environments and provide contextual cues [2,4]. As an immersive methodology, virtual reality (VR) is generally described as a fully immersive, interactive, and multisensory technology, where the 3D environments are generated by a computer and the whole experience is user-centred [19]. Generally, the virtual experience activates several sensory pathways by simultaneously promoting the action of the senses, which can lead to emotions and enhance the individual’s involvement [20]. From the perspective of sensory analysis and consumer perception, VR has fascinating applications, and according to the literature, it can be used in (i) the sensory analysis of food in different contexts, (ii) the evaluation of purchase behaviour, (iii) the analysis of the environment’s influence on eating behaviour, as well as (iv) the treatment of eating disorders in controlled environments [21]. Several studies analysing shopping behaviour demonstrated that VR was effective in recreating contexts [22,23,24]. This technology has also been considered a valuable tool in the sensory/emotional evaluation of food products, as it can provide results like those performed in real environments [8,25,26]. Virtual reality has also been used to recreate food products, and no significant differences in the evaluation of virtual and real products were reported [27]. Immersive environments can overcome traditional tests’ limitations by achieving control over environmental variables that are the same for all study participants and, simultaneously, with greater ecological validity than in a laboratory test [21].

However, despite these advantages, VR can hinder the participants’ ability to see and interact with the “real” product. Therefore, newer technologies such as mixed reality (MR) can overcome this flaw by combining real and virtual objects. More specifically, mixed reality can be divided into “Augmented Reality—AR”, where the virtual is overlaid on top of the real world, and “Augmented Virtuality—AV”, where real-world items are added to the virtual world [28,29]. Examples of AR technology are *Pokémon Go* and Microsoft HoloLens, where digital information is overlaid on top of physical reality. At the same time, with AV, participants are immersed in a virtual/digital environment but can still interact with elements in the real world which are augmented in the virtual environment [30]. AV has been less explored compared to VR or AR [28,29], and in the context of consumer perception and sensory evaluation, the use of MR systems such as AV is still relatively rare [25,31].

Both the scientific community and sensory evaluation and consumer perception companies face the challenge of increasing the effectiveness of applied methods to better comprehend consumers’ behaviours, attitudes, sensory perception, and ultimately, the success of new food products. The importance of contextual cues in food choices, perception, and preferences is already established and defined as a major factor for food-related behaviours. Still, the existing methods cannot guarantee an adequate immersion level and are not helpful in predicting consumer behaviours in the real world. The developed methods should guarantee rigorous scientific control (e.g., internal validity), the ability to be implemented with large consumer panels, and the provision of additional insights for members of the scientific community and actors in the agri-food sector.

Recognizing the relevance of these topics, this study focuses on evaluating the impact of context on food acceptance, using an augmented virtuality system that allows for the realistic recreation of environments. Thus, this work’s main goal was to develop an augmented virtuality (AV) system capable of being applied for the sensory evaluation of food products with untrained consumers. Furthermore, we sought to evaluate the impact of this technology on the hedonic evaluation of products and to analyse the effects of the environment itself on the level of engagement with the task. Finally, immersive environments with different levels of contextual cues were applied to assess how these differences could impact not only a hedonic evaluation but also engagement and presence levels.

## 2. Materials and Methods

### 2.1. Sensory Panel

A total of 102 participants were recruited from the sensory evaluation company Sense Test’s consumer database, a sensory evaluation and consumer testing company in Vila Nova de Gaia, Portugal. They were mainly residents of the Oporto metropolitan area in the north of Portugal. Data from three subjects were removed due to failure to attend or complete all three experimental sessions, resulting in a final sample of 99 subjects (female—n = 55, male—n = 44, average age—32.8 ± 8.0 years old). These participants were recruited based on their willingness to participate in a consumer test using an AV system. Participants were instructed to avoid eating, drinking, and smoking 1 h before participating in the sensory evaluation sessions. Participants also had to be regular consumers of the product under evaluation, peach nectars, and be under 50 years old. The limitation on age was applied to assure that eligible participants would be familiarised or at least comfortable with modern technologies and thus better equipped to perform sensory evaluation tests with AV. Although no questionnaire was applied to assess the familiarity level of the participants with VR technologies, during the explanation of the AV system to the participants (Section 2.3), they were asked about their previous experience with virtual reality, and the vast majority did not have any experience with this type of technology. The product to be evaluated was chosen based on its familiarity with Portuguese consumers, as the nectar flavour is the most consumed in Portugal (AIJN, 2018), and the ease of being manipulated and consumed under an AV system.

All participants received a small amount of financial compensation for their participation. The company ensures the protection and confidentiality of data through authorisation 2063/2009 [32] of the National Data Protection Commission and established internal conduct, following GDPR standards from European Regulation (EU) 2016/679 [33]. Moreover, informed consent was obtained, and participants could quit the evaluation anytime.

### 2.2. Samples

Five different types of commercial peach nectars, consisting of the most popular branded and private label products, were tested, and an effort was made to select products with differences in their sensory profiles. Samples were presented to consumers in their original packages (*ca.* 200 mL), coated with grey self-adhesive paper to avoid brand recognition, and were identified with a random three-digit code that was different across testing environments. The individual packaging for all samples was quite similar, with a popular brick shape with only slight variations in size, which prevented the participants from recognizing particular brands from the shape outline of the package. Samples were also divided according to their batches to ensure that each consumer tasted samples from the same batch in all tests. Furthermore, samples were kept refrigerated (4 °C) until they were presented to consumers. Each tasting session was approximately 20–30 min, and black cardboard straws were provided to consumers with each sample to consume the peach nectars. Additionally, participants were instructed not to drink the whole sample to avoid over-consumption and only to consume enough sample to perform the evaluations.

In all of the tests, samples were presented following a sequential monadic balanced order of presentation, according to the Latin square design, to counterbalance possible carry-over effects [34]. Across all test settings, participants were provided with a glass of bottled natural still water and unsalted crackers and were asked to chew a piece of cracker and rinse the mouth with water between each sample to cleanse the palate. Furthermore, participants were provided with a porcelain spittoon in the laboratory setting.

### 2.3. Testing Environments

Sensory evaluations were performed under three different conditions, varying in terms of the environment—a laboratory setting with no evoked context and an AV (AV) system recreating two environments: a public food court and a house living room. The environments used in the AV system were chosen to reflect real-life environments where the consumption of fruit nectars occurs. Furthermore, the environments presented several differences concerning contextual cues (e.g., sound and presence of other consumers).

#### 2.3.1. Laboratory Setting

**Figure 1:**
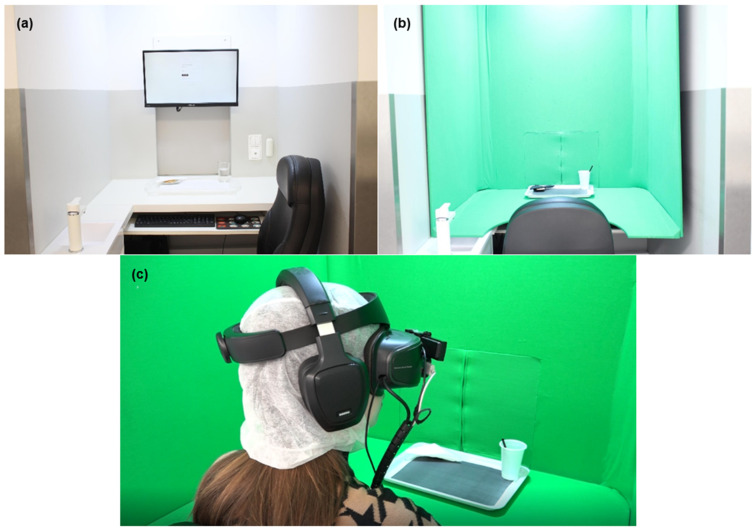
(**a**) The sensory booth setup for sensory evaluation in the laboratory setting. (**b**) The booth setup for the realisation of the test was performed with the AV system, with the sensory booth covered with green chroma key fabric. (**c**) A participant performing a sensory evaluation in the AV system.

For the laboratory setting, tests were performed in individual sensory booths at Sense Test’s sensory evaluation lab, equipped in accordance with ISO 8589:2007 [35] (Figure 1a). Room temperature (*ca*. 19 °C) was controlled during the sessions, and each sensory booth was under white lighting.

#### 2.3.2. Augmented Virtuality System

**Figure 2:**
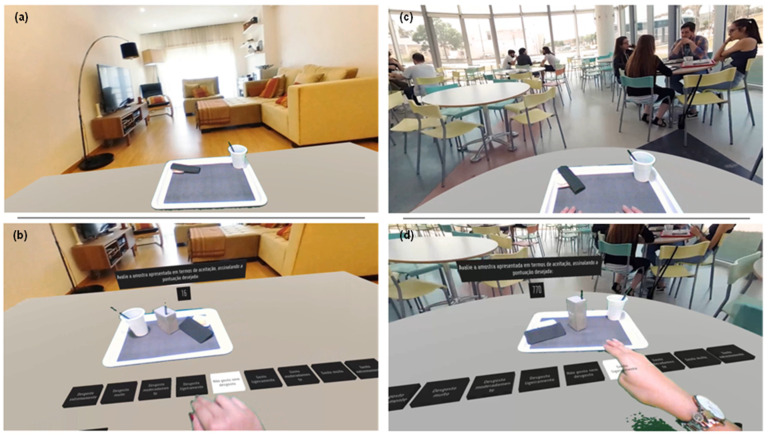
AV testing environments used in study. (**a**,**b**)—Virtual living room (living room_AV). (**c**,**d**)—Virtual public food court (public food court_AV).

This work used an AV system, meaning consumers were involved in a virtual environment but could interact with both the virtual and real worlds. To achieve this, Lenovo Explorer (Lenovo, Hong Kong, China) headsets equipped with a Genius 120-degree Ultra Wide Angle Full HD Conference Webcam (KYE Systems Corporation, Taipei, Taiwan) and a Leap Motion Controller (Ultraleap, Bristol, United Kingdom),a hand-tracking device that allows for interaction with the virtual environment, were used. Additionally, headphones were used to recreate the sounds of the virtual environments. Furthermore, since an AV system was being applied, the participants needed to observe a virtual environment and simultaneously interact with and observe physical elements of the real world (e.g., hands, samples, trays). For this purpose, the virtual environments were projected into green chroma key fabric, Falcon Eyes, that covered wooden structures in the sensory booths of Sense Test’s sensory evaluation lab (Figure 1). Both virtual environments used in this test—a living room and a public food court (Figure 2)—were obtained through 360° video footage of the locations. The sound was also recorded and incorporated into the sessions. In the living room, it was possible to hear a television as a surrounding sound, while in the public food court, the environmental sound of the real environment was used.

Consumers first calibrated the leap motion sensor for each test performed in this system to detect their hands. After the calibration, the virtual environment was projected for one minute without any instructions or questions to increase consumers’ immersion in the environment. After this period, the question and the sample number would appear in the eye line of consumers, and the 9-point hedonic scale was present on the virtual table (Figure 2b,d). Consumers could select the answer using their hands (choosing a button until it turned white) (Figure 2b,d). Data were collected automatically on SenseGest (Sense Test’s data collection software). The research team presented each sample after automatic instruction from the AV command systems without using any type of beacon or request by the participant. The technician responsible for the presentation of the samples wore a lab coat and gloves made of chroma key fabric to minimise the disruption in the virtual environment when samples had to be presented.

The first time consumers performed a test in this system, one of the research team members provided a brief explanation. This explanation was made with a demonstration video that did not have any of the environments used in this work. During the tests, the research team could monitor the consumers’ performance and provide any help when deemed necessary. Each test had a duration of about 20–30 min.

### 2.4. Sensory Evaluation

The tests were conducted between January and March 2020. Each participant used an individual balaclava protection under the headset. After each session, all individual equipment and the booth were thoroughly sanitised. Additionally, air purifiers were used to filter the air within the lab. A balanced design was applied to define the order in which consumers would evaluate the product in each setting. For each consumer, at least a one-week interval was imposed between sessions, with only one setting per session.

For each sample tested, consumers rated their overall liking on a classical 9-point hedonic scale, ranging from 1—dislike extremely to 9—like extremely [36]. After each session, consumers answered a 10-item Engagement Questionnaire (EQ) [14] on a 7-point scale ranging from “strongly disagree” to “strongly agree”. This questionnaire evaluated three factors: ‘Active Involvement’, ‘Purposeful Intent’, and ‘Affective Value’ (Table S1). For virtual environments, a 6-item Presence Questionnaire [37] was also answered on a 7-point scale, with scale anchors differing for each item (Table S1). Participants answered the questionnaires after being taken out of the immersed environments.

### 2.5. Data Analysis

All statistical analyses were performed using SPSS 27 software (IBM, Armonk, NY, USA). Data were reported as the average ± standard deviation (SD). To assess the effect of age on EQ and PQ scores, participants were divided into three groups according to the frequency distribution of age: group 1 (participants in the lowest quartile, age up to 26), group 2 (participants in the second and third quartile, age between 27 and 39), and group 3 (participants in the last quartile, age greater than 39). All analyses were performed at a 95% confidence level.

#### 2.5.1. Overall Liking

A 3-way mixed repeated measures ANOVA (environment as within-subject factor, participants and samples as between-subject factors) was initially performed for overall liking. Then, the data were grouped according to either the sample or environment. To assess how the environment affected the evaluation of each sample, 2-way mixed repeated measures ANOVAs (environment as within-subject factor, participants as between-subject factors) were applied to each sample. Then, to assess significant differences among samples in each environment, 2-way mixed ANOVAs (sample as fixed factor, participants as random factor) were applied to each environment. When the *p*-value from the Mauchly sphericity test was below 0.05, sphericity could not be assumed, and *F* values were determined through the Greenhouse–Geisser correction. Post hoc analysis was performed with Tukey’s *t*-test.

#### 2.5.2. Engagement and Presence

The reliability of the 10-item EQ and the 6-item PQ was assessed with Cronbach’s α. Sampling adequacy was evaluated with the KMO (Kaiser–Meyer–Olkin) test, with values above 0.6 indicating adequate sampling [38,39]. Reliability and sampling adequacy were assessed for each environment and session order.

For analysis of the EQ and PQ scores, a 3-way repeated measures ANOVA (environment as a within-subject factor; age and sex as between-subject factors) was performed for each EQ factor and presence score. Additionally, the data were grouped according to each environment, and 2-way ANOVAs (age and sex as fixed effects) were applied to each variable (EQ factors and presence scores) to assess the impact of socio-demographic characteristics in each environment. Sphericity and post hoc tests were performed as described above.

The correlation of the EQ factors across different environments was evaluated with Pearson’s correlation (2-tailed), following the work of Hannum, et al. [40]. The correlation between presence and EQ factor scores was also performed using Pearson’s correlation (2-tailed) for both AV environments and each AV environment.

## 3. Results

### 3.1. Overall Liking

Regarding the overall liking levels, samples significantly impacted liking scores, while the test environment did not significantly affect the overall liking scores (Table 1). Additionally, the interaction between the environment and the sample (*F* (7.756, 760.107) = 1.205; *p* = 0.294) had no significant effect on the liking levels.

Further analysis was performed for each sample and each environment separately (Table 2). Concerning the environment’s impact on the samples’ evaluation, the same sample’s evaluation was not significantly affected across the different environments. However, the environment influenced the discrimination of the samples, with the laboratory setting being the only one that significantly discriminated between the samples (*p* = 0.010).

### 3.2. Engagement

Cronbach’s α from the 10-item EQ showed that this questionnaire was reliable across all environments (α > 0.750) and session orders (α > 0.700, increasing from the first session to the last). The KMO result was also superior to 0.700 in all of the environments and session orders, showing acceptable sample adequacy (Table S2).

Regarding the different factors of the Engagement Questionnaire, differences across environments were reported for the ‘Purposeful Intent’ and ‘Affective Value’ factors (Table 3). For the ‘Purposeful Intent’ factor, the ‘public food court_AV’ environment had higher scores, while for the ‘Affective Value’ factor, higher scores were registered for both AV environments.

All correlations of the different EQ factors across environments were significant except for ‘Active Value’ between the laboratory and the ‘public food court_AV’. Furthermore, for all EQ factors, correlations between the two AV environments had higher values than correlations between any AV environment and the laboratory, particularly for the ‘Affective Value’ factor (Table 4).

Regarding socio-demographic characteristics, only age significantly affected the scores of the ‘Purposeful Intent’ factor of the EQ, with the older age group giving higher scores than the younger participants. Sex had no significant impact on any EQ factor scores (Table 5). Additionally, when evaluating each environment separately, the effects of age were only observed for evaluation in the ‘Laboratory’ setting, where the older group provided higher scores for the factors ‘Purposeful Intent’ (*p* = 0.007) and ‘Affective Value’ (*p* = 0.041) compared to the youngest group and females had higher scores for ‘Purposeful Intent’ (*p* = 0.026). For evaluations performed in AV environments, there were no differences between EQ factors among socio-demographic characteristics (Table S3).

### 3.3. Presence

Cronbach’s α from the 6-item Presence Questionnaire showed that the internal consistency was not severely influenced by the environment (*ca.* 0.650 for both AV environments). However, the session order substantially impacted the scale’s reliability, decreasing the value from the first evaluation (0.747) to the second evaluation (0.598). The KMO result was stable across environments and session orders (values between 0.650 and 0.699) (Table S4).

Concerning the levels of presence, the environment had a significant impact (*p* < 0.001) on the scores, with evaluations performed in the ‘public food court_AV’ having higher presence scores than those performed in the ‘living room_AV’. As it occurred with EQ factor scores, only age significantly impacted the overall presence scores, with the oldest group having higher scores than the youngest group (Table 6). When analysing each AV environment separately, it was possible to observe that this effect of age was only present for the evaluations performed in the ‘public food court_AV’ (Table S5).

Lastly, it was also possible to determine a positive correlation between presence scores and the scores from each EQ factor. This positive correlation was verified on an overall level and for each AV environment, with only minor differences between the correlation levels in each environment being reported (Table 7). However, it was possible to see that the highest correlations were verified for ‘Purposeful Intent’ in all of the tested conditions.

## 4. Discussion

### 4.1. Overall Liking

Several studies have reported that evaluations performed with evoked contexts or in real environments can lead to higher liking scores and higher discrimination between samples [2,4]. Concerning immersive environments, Bangcuyo, Smith, Zumach, Pierce, Guttman and Simons [13] and Hathaway and Simons [41] have reported higher product discrimination and several other works have reported higher liking scores [13,41,42,43,44,45]. However, the AV environments did not affect the participants’ liking of the samples in this work since overall liking scores were similar across environments, and the samples were evaluated equally across all environments. At the same time, hedonic discrimination only occurred in the laboratory setting (Table 2). Several studies have also reported only marginal effects of immersive environments on product liking and discrimination [46,47,48,49]. Similar to what is reported in this work, there have also been reports where immersive environments lead to lower discrimination between samples. This discrimination pertained to liking [40,50], intention to re-taste [51], emotional profile [25,51,52], and sensory profile [6]. Gouton, et al. [53] have also reported lower sample discrimination with VR than in sensory booths or with an evoked context. However, the credibility of the VR environments was higher than the credibility of the evoked context, and the discriminatory power of an environment is not representative of its external validity since the VR environment led to results comparable to those of the real environment.

This increase in the ability to discriminate between samples in the laboratory setting could be related to an increased focus by the consumers due to the lack of contextual cues [51], which makes consumers more prone to detect negligible differences between products [10]. In this study, an AV system was also used, increasing the participants’ immersion in the environments compared to other immersion technologies, such as virtual reality. This increased interaction with the environment could also explain the lack of discrimination between samples in the AV environments. It is also important to note that the effect of contextual cues is also very dependent on the context sensitivity of the product [8] and the congruency of the environment [54], with evaluations in congruent environments leading to higher liking scores [55,56] or heightened taste perception [57]. The product we used in this study may be independent of environmental cues, diluting the AV environments’ effect on hedonic responses. The possible incongruence between the tested environments, ‘public food court_AV’ and ‘living room_AV’, and the evaluated product could have also led to the lack of effect of the environments on product overall liking.

### 4.2. Engagement

To assess the level of consumers’ engagement with the tasks, the newly developed Engagement Questionnaire [14] was used. With this scale, engagement was divided into three factors: ‘Active Involvement’—thoughts and focus directed on the task throughout the entirety of the duration of the task, ‘Purposeful Intent’—perceived personal relevance of the task, and ‘Affective Value’—additional interest in the task.

In the present work, the environment significantly influenced the ‘Purposeful Intent’ and ‘Affective Value’ levels, with evaluations performed in AV environments, particularly in ‘public food court_AV’, leading to higher scores for these factors (Table 3). Previous research has reported that immersive environments lead to lower ‘Active Involvement’ compared to the laboratory setting and reported no significant differences in ‘Purposeful Intent’ and ‘Affective Value’ [40]. Nonetheless, the increases in ‘Purposeful Intent’ and ‘Affective Value’ caused by performing the sensory evaluations in AV environments are not surprising because other works have reported higher levels of consumer engagement and enjoyment with immersive environments [13,41,47,51]. For instance, Hannum, Forzley, Popper and Simons [40] speculated that the immersive environment used in their study did not affect ‘Purposeful Intent’ factor scores because the evaluated product, wine, was an intrinsically engaging stimulus. On the other hand, the product used in this study does not have strong cultural or social associations, and thus, the use of an AV environment causes an increase in ‘Purposeful Intent’ scores. As such, immersive environments can increase the relevance of the tasks when consumers evaluate products that are not engaging by nature. The verified increase in ‘Affective Value’ could also mean that consumers were distracted by the environment and the technology, which can further explain the lack of hedonic discrimination in the evaluations performed in both AV environments (Table 2). In future works, warm-up sessions with the technology could be performed to avoid potential feelings of excitement associated with AV [25]. Additionally, when consumers use the new technology for the first time, they might feel more predisposed to explore it instead of feeling immersed in the environment or behaving spontaneously [22].

Furthermore, the correlation of the factors across different environments was higher between the AV environments than between each AV environment, either ‘public food court_AV’ or ‘living room_AV’, and the laboratory setting (Table 4), indicating that consumers were more consistent in their evaluations across both AV environments. Hannum, Forzley, Popper and Simons [40] reported that the correlations of factors were stronger between the immersive and the real environment than between the immersive environment and the laboratory setting.

The socio-demographic characteristics also affected EQ factor scores, with the older group providing higher scores for ‘Purposeful Intent’ (Table 5). Furthermore, the effect of age was only present in the laboratory setting, with the older group having higher scores for ‘Purposeful Intent’ and ‘Affective Value’. These results could have occurred because the evaluation performed in the laboratory setting might be less exciting and more boring. Younger adults can be more prone to feel bored when performing tedious and monotonous tasks [58]. Interestingly, age-related differences were non-existent in the evaluations performed with AV (Table S3).

### 4.3. Presence

Regarding the level of presence, it was possible to report a higher score in the virtual environment for ‘public food court_AV’ than for ‘living room_AV’ (Table 6). There are several explanations for this difference, despite both environments being generated through 360° video recordings of real environments. The most plausible explanation is that the ‘public food court_AV’ visual and audio stimuli provided a more coherent environment for the consumers. In ‘public food court_AV’, the auditory stimuli included environmental sounds from the real environment (e.g., people talking, sounds of plates and silverware), while for ‘living room_AV’, only the sound of a television in the background was provided. Furthermore, in the ‘public food court_AV’ environment, there was also an element of a social environment since it was possible to observe other people eating and talking (Figure 2). The combination of increased multi-sensory stimuli and social cues could have increased the level of presence in the ‘public food court_AV’ virtual environment, and that environment could have been deemed more coherent than ‘living room_AV’ [59,60,61,62]. Furthermore, using an AV system where consumers could see and interact with elements of both the virtual and the physical world (Figure 2) could have naturally increased the level of presence compared to the simple utilisation of an immersive environment created with a virtual reality [28]. Lastly, ‘public food court_AV’ can be a more generic and familiar environment for the participants, while ‘living room_AV’ is more personal, which leads to lower presence scores. Besides these effects on presence levels, using more personally relevant environments can lead to more repeatable data and reliable insights [61]. Additionally, there was a significant correlation between presence level and EQ factor scores (Table 7), further highlighting the importance of immersive environments’ quality in participants’ engagement and enjoyment [63,64]. Previously, Colla, et al. [65] have also demonstrated that a consumer’s emotional status (e.g., pleasure and arousal) positively influences the sense of presence.

A similar effect to the EQ factors was observed regarding socio-demographic characteristics, with the older group having higher presence scores (Table 6). Xu, et al. [66] also reported a significant and positive correlation between the presence level in an AV environment and age. Additionally, when analysing each AV environment separately, the effect of age was only observed for ‘public food court_AV’, although for the ‘living room_AV’ environment, the older group also had the highest presence scores (Table S5). The lack of effect of sex is not particularly surprising since there are conflicting reports in the literature. Although increased levels of presence in virtual environments are more associated with males—particularly in more complex tasks [67,68]—females have also been reported to have higher levels of presence, especially in more straightforward tasks [68].

## 5. Limitations

In this study, an AV system for the performance of sensory evaluation was developed and validated. Due to the novelty of the technology and the scope of this study, some limitations should be mentioned.

Firstly, the sensory panel does not represent the overall population since an age limit was applied. In the preliminary tests performed with the AV system, it was observed that older participants felt uncomfortable with new technologies and had difficulty performing the tasks. This may represent a problem associated with the use of immersive technologies since this segment of the population might not be capable of performing the tasks, or participants might be distracted by the technology and, as such, not be immersed in the environment or not be mindful of the food product during the evaluation. Although an age limit was imposed in this study, it was possible to observe that the older participants already showed differences in engagement and presence level.

Another possible limitation of this study is that only the socio-demographic variables were considered to assess their impact on engagement and presence scores. Other variables, such as experience with immersive technologies, also impact engagement and presence. Although this information was not registered, most participants mentioned having no experience with virtual or AV systems while explaining the AV system (Section 2.3.2). However, the effects of age on engagement and presence could have been caused by increased familiarity with video games or movies among the younger groups, which can decrease the feeling of novelty when experiencing the AV system in this work. Yang, Nijman, Flintham, Tennent, Hidrio and Ford [62] have previously reported that training sessions can be necessary to decrease the novelty associated with immersive environments and its potential effects on product evaluation or attitudes toward the task.

The products and the environments tested could also constitute another study limitation. The lack of effect of the virtual environments on the hedonic evaluations of the peach nectars could have been caused by the incongruence between the environments and the tested product. Another possible explanation is that the tested product is context-insensitive; thus, contextual cues do not affect product evaluation. To confirm this assumption, a previous questionnaire could have been applied to assess if participants associated peach nectars with specific environments or to determine the most regular consumption environments of peach nectars. However, the food product and environments were chosen considering that the primary goal of this work was to develop and validate the AV system and that most participants did not have experience with this type of technology. As such, the product was chosen due to its ease of manipulation and consumption, while the environments reflected two locations where the consumption of peach nectar might occur while also presenting contextual differences.

Lastly, as this study’s goal was to develop an AV system capable of being applied for the sensory evaluation of food products with untrained consumers, the authors did not perform any external validation related to the ecological validity of the methods used. In follow-up studies, a comparison between how foods are perceived in a real context and how they are perceived in different settings (e.g., sensory booths and developed AV systems) should be performed. The developed AV system should also be compared with other immersive environments, such as virtual reality, augmented reality, or a video wall/LED wall.

## 6. Conclusions

The main goal of this study was achieved since a panel of untrained consumers could perform sensory evaluations of food products using the AV system. Although the test environments did not impact product evaluations, evaluations in sensory booths presented higher discriminatory power. Nevertheless, the developed AV system increased the level of engagement with the task, which was correlated with the sense of presence in the virtual environments. As such, this study further demonstrates the advantages of using immersive technologies for the sensory evaluation of food products, and in particular, it shows that untrained consumers can accurately perform evaluations with an AV system. However, further studies should include evaluations with more context-sensitive products and compare the virtual environments with the real environment to further validate the results obtained with the AV system. Moreover, it is also essential to assess if consumers can perform sensory and emotional profiling within the AV system.

## Figures and Tables

**Table 1:** Mean overall liking (±S.D) according to samples and test environment. *F* values from 3-way mixed repeated measures ANOVA (environment as within-subject factor, participants and samples as between-subject factors). a,b—homogeneous groups in accordance with Tukey’s test (*p* < 0.050).

Factor		Overall Liking	*F* (df_(factor, error)_); *p*
**Sample**	A	7.2 ^a^ (±1.6)	*F* (4, 392) = 2.816; *p* = 0.025
	B	7.0 ^a,b^ (±1.4)	
	C	7.0 ^a,b^ (±1.5)	
	D	6.8 ^a,b^ (±1.5)	
	E	6.8 ^b^ (±1.6)	
**Environment**	Laboratory	7.0 (±1.7)	*F* (1.939, 760.107) = 0.039; *p* = 0.958
	Public food court_AV	7.0 (±1.5)	
	Living room_AV	7.0 (±1.5)	

**Table 2:** Mean overall liking (±S.D) of peach nectar samples in each environment. *F* values from 2-way mixed repeated measures ANOVA (environment as within-subject factor, participants as between-subject factor) on overall liking and 2-way mixed ANOVA (sample as fixed factor, participants as random factor) on overall liking. a,b—homogeneous groups in each environment (**vertically**) in accordance with Tukey’s test (*p* < 0.050) for each environment.

Sample	Laboratory	Public Food Court_AV	Living Room_AV	*F* (df_(environment, error)_); *p*
A	7.2 ^a^ (±1.6)	7.1 ^a^ (±1.6)	7.2 ^a^ (±1.6)	*F* (2, 196) = 0.167; *p* = 0.847
B	7.0 ^a,b^ (±1.4)	7.0 ^a^ (±1.4)	7.0 ^a^ (±1.4)	*F* (2, 196) = 0.045; *p* = 0.956
C	7.2 ^a^ (±1.5)	6.9 ^a^ (±1.6)	7.0 ^a^ (±1.5)	*F* (2, 196) = 1.870; *p* = 0.157
D	6.8 ^a,b^ (±1.8)	6.9 ^a^ (±1.4)	6.8 ^a^ (±1.3)	*F* (2, 196) = 0.330; *p* = 0.719
E	6.6 ^b^ (±1.5)	6.9 ^a^ (±1.3)	7.0 ^a^ (±1.5)	*F* (2, 196) = 2.333; *p* = 0.100
***F*** **(df_(sample, error)_); *p***	*F* (4, 392) = 3.384; *p* = 0.010	*F* (4, 392) = 0.677; *p* = 0.608	*F* (4, 392) = 1.407; *p* = 0.731	

**Table 3:** Mean scores (±S.D) of each Engagement Questionnaire (EQ) factor for each environment. *F* values from 3-way repeated measures ANOVA (environment as within-subject factor; age and sex as between-subject factors) for each Engagement Questionnaire factor. a,b—homogeneous groups between environments (**horizontally**) in accordance with LSD (least significant difference) test (*p* < 0.050).

EQ Factor	Laboratory	Public Food Court_AV	Living Room_AV	*F* (df_(environment, error)_); *p*
Active Involvement	17.3 ^a^ (±3.7)	17.2 ^a^ (±3.6)	16.5 ^a^ (±3.9)	F (2, 186) = 1.276; *p* = 0.286
Purposeful Intent	25.6 ^b^ (±2.4)	26.0 ^a^ (±2.1)	25.7 ^b^ (±2.4)	F (2, 186) = 3.154; *p* = 0.045
Affective Value	16.4 ^b^ (±3.1)	18.5 ^a^ (±2.8)	18.0 ^a^ (±3.0)	F (1.712, 157.495) = 23.126; *p* < 0.001

**Table 4:** Engagement Questionnaire factor correlations across different environments, according to Pearson’s correlation.

**Active Involvement**	**Laboratory**	**Public Food Court_AV**	**Living Room_AV**
Laboratory	1	---	---
Public food court_AV	0.162	1	---
Living room_AV	0.254 *	0.407 **	1
**Purposeful Intent**	**Laboratory**	**Public Food Court_AV**	**Living Room_AV**
Laboratory	1	---	---
Public food court_AV	0.456 **	1	---
Living room_AV	0.516 **	0.585 **	1
**Affective Value**	**Laboratory**	**Public Food Court_AV**	**Living Room_AV**
Laboratory	1	---	---
Public food court_AV	0.356 **	1	---
Living room_AV	0.242 **	0.628 **	1

*—Correlation significant at 0.05 level; **—correlation significant at 0.01 level.

**Table 5:** Mean scores (±S.D) of each Engagement Questionnaire (EQ) factor according to socio-demographic characteristics. *F* values from 3-way repeated measures ANOVA (environment as within-subject factor; age and sex as between-subject factors) for each EQ factor. a,b—homogeneous groups (**horizontally**) according to Tukey’s test (*p* < 0.050).

**AGE**				
**EQ Factor**	**18–26** **(n = 28)**	**27–39** **(n = 46)**	**40–45** **(n = 25)**	***F*** **(df_(age, error)_); *p***
Active Involvement	16.2 ^a^ (±4.0)	17.0 ^a^ (±3.5)	17.8 ^a^ (±3.8)	*F* (2, 93) = 2.441; *p* = 0.093
Purposeful Intent	25.1 ^b^ (±2.5)	25.7 ^ab^ (±2.4)	26.5 ^a^ (±1.6)	*F* (2, 93) = 4.172; *p* = 0.018
Affective Value	17.2 ^a^ (±3.5)	17.6 ^a^ (±3.0)	18.1 ^a^ (±2.6)	*F* (2, 93) = 1.068; *p* = 0.348
**SEX**				
**EQ Factor**	**Female** **(n = 54)**	**Male** **(n = 45)**		***F*** **(df_(sex, error)_); *p***
Active Involvement	17.2 (±3.6)	16.7 (±3.9)		*F* (1, 93) = 1.106; *p* = 0.296
Purposeful Intent	26.1 (±2.1)	25.4 (±2.5)		*F* (1, 93) = 2.546; *p* = 0.114
Affective Value	17.7 (±2.9)	17.5 (±3.3)		*F* (1, 93) = 0.012; *p* = 0.912

**Table 6:** Mean scores (±S.D) of presence according to environment, age, and sex. *F* values from 3-way repeated measures ANOVA (environment as within-subject factor; age and sex as between-subject factors) for presence scores. a,b—significantly different results (**horizontally**) between environments (*p* < 0.050).

**Environment**	**Public Food Court_AV**	**Living Room_AV**		***F*** **(df_(environment, error)_); *p***
	31.4 ^a^ (±6.2)	27.6 ^b^ (±6.6)		F (1, 93) = 39.711; *p* < 0.001
**Age**	**18–26** (n = 28)	**27–39** (n = 46)	**40–45** (n = 25)	**F (df_(age, error)_); *p***
	27.8 ^b^ (±7.5)	29.4 ^a,b^ (±5.8)	31.6 ^a^ (±6.8)	F (1, 93) = 3.711; *p* = 0.028
**Sex**	**Female** (n = 54)	**Male** (n = 45)		**F (df_(sex, error)_); *p***
	30.2 (±6.4)	28.7 (±6.9)		F (1, 93) = 2.349; *p* = 0.129

**Table 7:** The correlation between Presence Questionnaire scores and Engagement Questionnaire factors on an overall level and for each AV environment, according to Pearson’s correlation.

Presence	Active Involvement	Purposeful Intent	Affective Value
Overall	0.385 **	0.506 **	0.405 **
Public food court_AV	0.319 **	0.502 **	0.380 **
Living room_AV	0.429 **	0.516 **	0.420 **

**—correlation significant at 0.01 level.

## Data Availability

The data presented in this study are available on request from the corresponding author due to consumer data privacy.

## Supplementary Materials

The following supporting information can be downloaded at https://www.mdpi.com/article/10.3390/foods13152456/s1, Table S1: Items composing Engagement [14] and Presence (adapted from [34]) Questionnaires. Table S2: Reliability (Cronbach’s α) and sampling adequacy (KMO) of 10-item Engagement Questionnaire across environments and session orders. Table S3: Mean scores (±S.D) of each Engagement Questionnaire factor according to age groups and sex for each augmented virtuality environment. *F* values from 2-way ANOVA (age and sex as fixed factor) for each Engagement Questionnaire factor in each environment, a,b—homogeneous groups according to Tukey’s test (*p* < 0.050). Table S4: Reliability (Cronbach’s α) and sampling adequacy (KMO) of 6-item Presence Questionnaire across augmented virtuality environments and evaluation orders. Table S5: Mean scores (±S.D) of presence according to age groups and sex for each augmented virtuality environment. *F* values from 2-way ANOVA (age and sex as fixed factor) for presence scores in each environment, a,b—homogeneous groups according to Tukey’s test (*p* < 0.050).
