# Supplementary material for: Sensory Analysis Performed within Augmented Virtuality System: Impact on Hedonic Scores, Engagement, and Presence Level
Source: Foods. 2024 Aug 3;13(15):2456. doi: 10.3390/foods13152456 (PMC11311452; doi:10.3390/foods13152456)
Supplement: Supplementary file 1 [file foods-13-02456-s001.zip › foods-3123176-supplementary.pdf]

Supplementary material to:

Sensory Analysis Performed within Augmented Virtuality System: Impact on Hedonic Scores, Engagement, and Presence Level

José Carlos Ribeiro [1], Célia Rocha [1,2], Bruna Barbosa [1,2], Rui Costa Lima [2] and Luís Miguel Cunha [1,*]

[1] GreenUPorto/INOV4Agro & DGAOT, Faculty of Sciences of the University of Porto, Rua da Agrária, 747, 4485-646 Vairão, Portugal;
[2] Sense Test, Lda, Rua Zeferino Costa, 341, 4400-345 Vila Nova de Gaia, Portugal;
* Correspondence: lmcunha@fc.up.pt; Tel.: +351-252660400

Table S1. Items composing the Engagement (Hannum & Simons, 2020) and Presence (adapted from Usoh, Catena, Arman, and Slater (2000)) questionnaires.

	Engagement	Presence (scale anchors)
**Active Involvement**	I felt myself zoning out during the task. (R)	I had the sensation of being in the virtual environment. (1 - Very little; 7 - Completely)
	I lost interest in the task. (R)	There were times during the experience when the virtual environment was the reality for me (1 – Never; 7 – Almost always)
	I was distracted. (R)	When you think back to the experience, do you think of the virtual environment more as…(1 – Images I’ve seen; 7 – Places I visited)
**Purposeful Intent**	I felt dedicated to finish the task.	During the time of the experience, the strongest feeling was… (1 – Be inside the virtual environment; 7 – Be somewhere else)
	My contribution was significant to the outcome of the task.	Consider your memory of being in the virtual environment, how similar it is to places you have visited recently? Consider aspects such as realism, size, colors… (1 – Not similar; 7 – Very similar)
	I wanted to devote my full attention to the task.	During the time of your experience, I thought that I were actually in the virtual environment… (1 – Not frequently; 7 – Very frequently)
	I found the task meaningful.	

Engagement		Presence (scale anchors)
**Affective Value**	During the task, I was enjoying myself.	
	I found the task captivating.	
	I was motivated to expend extra effort during the task.	

Hannum, M. E., & Simons, C. T. (2020). Development of the engagement questionnaire (EQ): A tool to measure panelist engagement during sensory and consumer evaluations. *Food Quality and Preference, 81*, 103840. doi:https://doi.org/10.1016/j.foodqual.2019.103840

Usoh, M., Catena, E., Arman, S., & Slater, M. (2000). Using Presence Questionnaires in Reality. *Presence: Teleoperators and Virtual Environments, 9*. doi:10.1162/105474600566989

Table S2. Reliability (Cronbach's α) and sampling adequacy (KMO) of the 10-item Engagement Questionnaire across environments and session order.

Environment	Cronbach's α	KMO
Laboratory	0.771	0.792
Public dining area	0.777	0.835
Living room	0.751	0.750
**Session order**	**Cronbach's α**	**KMO**
1	0.730	0.806
2	0.729	0.756
3	0.820	0.852

Table S3. Mean scores (± S.D) of each Engagement Questionnaire Factor according to age groups and sex, for each Augmented Virtuality environment. F values from 2-Way ANOVA (age and sex as fixed factors) for each Engagement Questionnaire Factor in each environment a,b – homogeneous groups according to Tukey's test (p < 0.050)

Environment	**Engagement Questionnaire Factor**	**18-26** (n = 28)	**27-39** (n = 46)	**40-45** (n = 25)	F (df$_{(\text{age, error})}$); p
**Laboratory**	Active Involvement	16.0 (± 4.1)	17.7 (± 3.3)	17.8 (± 3.7)	F (2, 99) = 2.466; p = 0.090
	Purposeful Intent	24.5^{b} (± 2.8)	25.8^{ab} (± 2.4)	26.5^{a} (± 1.6)	F (2, 99) = 5.241; p = 0.007
	Affective Value	15.1^{b} (± 3.9)	16.8^{ab} (± 2.5)	17.1^{a} (± 2.7)	F (2, 99) = 3.295; p = 0.041
	**Engagement Questionnaire Factor**	**F** (n = 54)	**M** (n = 45)		F (df$_{(\text{sex, error})}$); p
	Active Involvement	17.7 (± 3.6)	16.8 (± 3.9)		F (1, 99) = 2.033; p = 0.157
	Purposeful Intent	26.0 (± 2.2)	25.0 (± 2.6)		F (1, 99) = 3.411; p = 0.068
	Affective Value	16.6 (± 3.1)	16.2 (± 3.1)		F (1, 99) = 0.073; p = 0.787
**Public food court**	**Engagement Questionnaire Factor**	**18-26** (n = 28)	**27-39** (n = 46)	**40-45** (n = 25)	F (df$_{(\text{age, error})}$); p
	Active Involvement	17.0 (± 3.9)	16.9 (± 3.2)	17.8 (± 4.1)	F (2, 99) = 0.548; p = 0.580
	Purposeful Intent	25.9 (± 1.8)	25.6 (± 2.5)	26.8 (± 1.5)	F (2, 99) = 2.290; p = 0.107
	Affective Value	18.5 (± 2.7)	18.2 (± 3.2)	18.9 (± 2.4)	F (2, 99) = 0.621; p = 0.540
	**Engagement Questionnaire Factor**	**F** (n = 54)	**M** (n = 45)		F (df$_{(\text{sex, error})}$); p
	Active Involvement	17.0 (± 3.8)	17.4 (± 3.4)		F (1, 99) = 0.273; p = 0.603
	Purposeful Intent	26.1 (± 2.0)	25.8 (± 2.3)		F (1, 99) = 0.326; p = 0.570
	Affective Value	18.6 (± 2.5)	18.3 (± 3.2)		F (1, 99) = 0.143; p = 0.706
**Living room**	**Engagement Questionnaire Factor**	**18-26** (n = 28)	**27-39** (n = 46)	**40-45** (n = 25)	F (df$_{(\text{age, error})}$); p
	Active Involvement	15.7 (± 4.1)	16.4 (± 3.8)	17.7 (± 3.7)	F (2, 99) = 1.953; p = 0.148
	Purposeful Intent	24.8 (± 2.7)	25.8 (± 2.4)	26.4 (± 1.8)	F (2, 99) = 3.064; p = 0.052
	Affective Value	17.9 (± 3.0)	17.8 (± 3.2)	18.4 (± 2.5)	F (2, 99) = 0.360; p = 0.619
	**Engagement Questionnaire Factor**	**F** (n = 54)	**M** (n = 45)		F (df$_{(\text{sex, error})}$); p
	Active Involvement	16.9 (± 3.5)	16.0 (± 4.3)		F (1, 99) = 1.770; p = 0.187
	Purposeful Intent	26.0 (± 2.2)	25.3 (± 2.6)		F (1, 99) = 2.001; p = 0.155
	Affective Value	18.0 (± 2.7)	18.0 (± 3.4)		F (1, 99) = 0.143; p = 0.706

Table S4. Reliability (Cronbach's α) and sampling adequacy (KMO) of the 6-item Presence questionnaire across Augmented Virtuality environments and evaluation order.

Environment	Cronbach's α	KMO
Public food court	0.670	0.665
Living room	0.650	0.650
**Evaluation order**	**Cronbach's α**	**KMO**
1	0.747	0.699
2	0.598	0.689

Table S5. Mean scores (± S.D) of Presence according to age groups and sex, for each Augmented Virtuality environment. F values from 2-Way ANOVA (age and sex as fixed factors) for presence scores in each environment. a,b – homogeneous groups according to Tukey's test (p < 0.050)

Factor		Public food court	Living room
Age (years)	18-26 (n = 28)	30.1^{b} (± 6.9)	25.4^{a} (± 7.4)
	27-39 (n = 46)	30.7^{ab} (± 5.8)	28.1^{a} (± 5.5)
	40-45 (n = 25)	34.0^{a} (± 5.6)	29.2^{a} (± 7.2)
**F ($df_{(age, error)}$); p**		F (2, 99) = 3.352; p = 0.039	F (2, 99) = 2.858; p = 0.062
Sex	M (n = 45)	31.0 (± 6.5)	26.4 (± 6.6)
	F (n = 54)	31.7 (± 6.0)	28.6 (± 6.5)
**F ($df_{(sex, error)}$); p**		F (1, 99) = 0.878; p = 0.351	F (1, 99) = 2.870; p = 0.094
